# Association of alpha‐thalassemia and Glucose‐6‐Phosphate Dehydrogenase deficiency with transcranial Doppler ultrasonography in Nigerian children with sickle cell anemia

**DOI:** 10.1002/jcla.23802

**Published:** 2021-05-03

**Authors:** Oyesola Oyewole Ojewunmi, Titilope Adenike Adeyemo, Ajoke Idayat Oyetunji, Yewande Benn, Mfoniso Godwin Ekpo, Bamidele Abiodun Iwalokun

**Affiliations:** ^1^ Sickle Cell Foundation Nigeria Surulere, Lagos Nigeria; ^2^ School of Cancer and Pharmaceutical Sciences King's College London London UK; ^3^ Department of Haematology & Blood Transfusion College of Medicine University of Lagos Lagos Nigeria; ^4^ Department of Biotechnology Afe Babalola University Ado‐Ekiti Nigeria; ^5^ Department of Molecular Biology and Biotechnology Nigerian Institute of Medical Research Lagos Nigeria

**Keywords:** alpha‐thalassemia, glucose‐6‐phosphate dehydrogenase deficiency, sickle cell anemia, stroke, transcranial Doppler ultrasonography

## Abstract

**Background:**

Stroke is a devastating complication of sickle cell anemia (SCA) and can be predicted through abnormally high cerebral blood flow velocity using transcranial Doppler Ultrasonography (TCD). The evidence on the role of alpha‐thalassemia and glucose‐6‐phosphate dehydrogenase (*G6PD*) deficiency in the development of stroke in children with SCA is conflicting. Thus, this study investigated the association of alpha‐thalassemia and G6PD(A^−^) variant with abnormal TCD velocities among Nigerian children with SCA.

**Methods:**

One hundred and forty‐one children with SCA were recruited: 72 children presented with normal TCD (defined as the time‐averaged mean of the maximum velocity: < 170 cm/s) and 69 children with abnormal TCD (TAMMV ≥ 200 cm/s). Alpha‐thalassemia (the α‐3.7 globin gene deletion) was determined by multiplex gap‐PCR, while G6PD polymorphisms (202G > A and 376A > G) were genotyped using restriction fragment length polymorphism—polymerase chain reaction.

**Results:**

The frequency of α‐thalassemia trait in the children with normal TCD was higher than those with abnormal TCD: 38/72 (52.8%) [α‐/ α α: 41.7%, α ‐/ α ‐: 11.1%] versus 21/69 (30.4%) [α‐/ α α: 27.5%, α ‐/ α ‐: 2.9%], and the odds of abnormal TCD were reduced in the presence of the α‐thalassemia trait [Odds Ratio: 0.39, 95% confidence interval: 0.20–0.78, *p* = 0.007]. However, the frequencies of G6PDA^−^ variant in children with abnormal and normal TCD were similar (11.6% vs. 15.3%, *p* = 0.522).

**Conclusion:**

Our study reveals the protective role of α‐thalassemia against the risk of abnormal TCD in Nigerian children with SCA.

## Graphical abstract

We investigated the association of alpha‐thalassemia deletion and G6PD deficiency [G6PDA‐] with stroke risk as stratified by TCD velocity status among Nigerian children with sickle cell anemia. Our results show that absence of α‐thalassemia increased risk of abnormal TCD and remains an independent predictor of abnormal TCD alongside lower hemoglobin oxygen saturation.

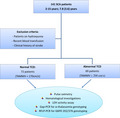

## 1 INTRODUCTION

Stroke is a severe complication of sickle cell anemia which may result in a permanent neurocognitive impairment and physical dysfunction.^1^ Although the risk of stroke persists throughout the entire life of persons living with SCA, it is more likely in the first decade of life.^2^, ^3^ The incidence of ischemic stroke is 1.02% per year between 2 and 5 years old while hemorrhagic stroke is predominant between 20–29 years of age; the latter is believed to be caused by stenosis of major intracranial vessels leading to moya‐moya or by the formation of aneurysms in the vessels of the circle of Willis.^4^, ^5^ An ischemic stroke usually results from occlusion of large cerebral arteries with an interplay of several mechanisms involved in hemolysis, hypoxia, anemia, abnormal red blood cell rheology, inflammation, endothelial dysfunction, and genetic factors.^6^ Previous studies have also identified prior transient ischemic attack, anemia, a past history or increased frequency of acute chest syndrome, and elevated systolic blood pressure as independent predictors of ischemic stroke, while anemia and high white blood cell count were also found to be independently associated with hemorrhagic stroke.^1^, ^4^

Significant progress has been made in the prevention of first stroke occurrence with the use of transcranial Doppler (TCD) screening for children with sickle cell anemia. The TCD scan can assist in identifying children with abnormal TCD velocity by measuring the elevated time‐averaged mean of maximum velocity in the large intracranial vessels of the circle of Willis, specifically in the middle cerebral and internal carotid arteries. The use of TCD has been recommended as a stroke risk screening tool for children with SCA to enable prompt identification of children with abnormally high TCD velocities.^7^ This is measured as time‐averaged mean of the maximum velocity: TAMMV > 200 cm/s. Children with SCA with normal TCD have TAMMV values below 170 cm/s. TCD scan provides an opportunity to target children with abnormally high TCD velocities for prophylactic interventions with hydroxyurea and blood transfusion.^7^ Unlike chronic blood transfusion which is fraught with many challenges in low resource countries, hydroxyurea therapy has been shown to be effective for primary stroke prevention in Nigerian children with SCA.^8^, ^9^

Alpha‐thalassemia is characterized by deletional loss of one or both alpha‐globin genes. α‐3.7 kb thalassemia deletion is the most common alpha‐thalassemia deletion in sub‐Sahara African populations and is a well‐known genetic modifier of SCA. Varied prevalence of α‐thalassemia deletion (‐α^3.7^) has been reported from different populations among persons with SCA: 30% in Brazil, 37% in Cameroon, 41–43% in Nigeria, 48% in France, and 56% in Tanzania.^10^, ^11^, ^12^, ^13^, ^14^, ^15^ Co‐inheritance of α^3.7^‐thalassemia with SCA is associated with increased risk of avascular necrosis and vaso‐occlusive crises while it improves survival, reduces rate of leg ulcers, the need for blood transfusion, and stroke risk.^11^, ^16^, ^17^ Some studies suggest that the protective effect of alpha‐thalassemia against stroke and abnormal TCD velocity may be related to the reduced hemolytic rate and improved rheological properties of red blood cells.^14^, ^18^

Glucose‐6‐phosphate dehydrogenase (G6PD) is an important source of Nicotinamide adenine dinucleotide phosphate (NADPH) in pentose phosphate pathway and is present in both erythrocytes and endothelial cells.^19^, ^20^ G6PD deficiency participates in increased generation of reactive oxygen species and decreased nitric oxide (NO) bioavailability and its impact on SCA may worsen vascular dysfunction and contribute significantly to cerebral vasculopathy.^20^, ^21^ *G6PD* deficiency is common in sub‐Saharan Africa and the predominant alleles: 202A and 376G as G6PDA^−^ are responsible only for approximately 12% of enzyme activity compared to the wild‐type 202G and 376A G6PD B allele.^22^ It has been suggested that *G6PD* deficiency worsens anemia and is linked to the increased need for blood transfusion, playing a significant role in the pathophysiology of cerebral vasculopathy.^23^ Findings on the association of alpha‐thalassemia deletion and *G6PD* deficiency with abnormal TCD velocity in children with sickle cell anemia from different populations are conflicting, suggestive of geographical and genetic modifiers.^13^, ^14^, ^24^, ^25^, ^26^

Despite the high burden of stroke risk and stroke in Nigerian SCA children, data on the clinical importance of alpha‐thalassemia and G6PD deficiency are limited. Here, we investigated the association of alpha‐thalassemia deletion and G6PD deficiency [202 G > A: (rs1050828) and 376 A > G (rs1050829) with stroke risk among Nigerian children with sickle cell anemia stratified by non‐imaging TCD velocities.

## 2 MATERIALS AND METHODS

### 2.1 Study design

In this study, we enrolled 141 children with SCA (2–15 years old) attending the Sickle Cell Foundation Nigeria, Lagos for TCD screening as part of their routine care over a 6 month period. The sickle cell anemia status of each enrollee was confirmed by alkaline electrophoresis to determine the hemoglobin SS (Hb SS) phenotype and solubility test to ascertain sickling characteristics of Hb SS in a deoxy‐state following the standard quality control procedures. Other inclusion criteria included being in a stable condition and not more than 16 years old at enrollment. Children who were not in steady‐state at presentation, aged >16 years old, had hemoglobin C phenotype, on hydroxyurea, received blood transfusion in the previous 4 months, or had clinical history of stroke were excluded from this study. Also excluded from the study were children with intermediate (conditional) stroke risk characterized by TAMMV of 170–199 cm/s.

### 2.2 TCD screening

Non‐imaging TCD was measured for all the children according to the Stroke prevention trial (STOP) guidelines as previously described^27^ and the time‐averaged mean of maximum velocities (TAMMV) were recorded. Children with TAMMV < 170 cm/s were defined as having normal TCD and represented a low stroke risk group, while children with TAMMV > 200 cm/s were defined as having abnormal TCD and represented high stroke risk group.^27^

### 2.3 Pulse oximetry and laboratory analyses

Pulse oximeter was used to measure hemoglobin oxygen saturation ​(SpO_2_) of the children after TCD scan. Full blood counts were determined using an automated hematology analyzer (Mindray, BC‐2800, China). Fetal hemoglobin was evaluated by high‐performance liquid chromatography (D‐10 Hemoglobin testing system, Bio‐Rad Laboratories, Inc., Hercules, CA, USA). Lactate dehydrogenase (LDH) activity was assayed spectrophotometrically at 340 nm with a Cobas kit (Roche/Hitachi analyzer, Roche Diagnostics GmbH). Percentage reticulocyte count was determined microscopically using blood films pre‐stained with new methylene blue.

### 2.4 Genetic analyses

A 3.7 kb alpha‐globin gene deletion was genotyped by multiplex gap‐PCR^28^ while glucose‐6‐phosphate dehydrogenase polymorphisms (202G > A and 376A > G) were genotyped using restriction fragment length polymorphism (RFLP‐PCR).^29^ Children with one (heterozygous) or two (homozygous) α‐globin gene deletions were regarded as having alpha‐thalassemia trait while they were classified as G6PD deficient (G6PDA^−^) or normal (G6PDA or G6PD B) based on the presence or absence of 202A/376G genotype.

### 2.5 Ethical considerations

Ethical approval was obtained from the Lagos University Teaching hospital health research and ethics committee (ADM/DCST/HREC/843). Written informed consent from parents/guardians and assent of the children who were 7 years old and above were obtained before enrolment.

### 2.6 Statistical analysis

Data obtained were summarized in percentages and mean ± standard deviation. The frequencies of the wild (G6PD202A/376G) and deficient (G6PD202G/376A) G6PD, as well as the alpha‐thalassemia trait observed, characterized by heterozygous and homozygous deletions (α α /‐ α and ‐ α /‐ α) versus normal α‐globin condition (α α / α α), were determined by counting the corresponding DNA bands on agarose gels. Categorical variables between the abnormal TCD and normal TCD groups were compared by chi‐square (χ^2^) and Fisher's exact tests, while differences between continuous variables were evaluated using Student's *t* test. Both the odds ratio (OR) and 95% confidence interval (95%CI) were also calculated. Multivariable logistic regression was also performed to determine independent predictor(s) of abnormal TCD. All statistical analyses were carried out using SPSS version 25 for Windows. Statistical outcomes with *p*‐value <0.05 were considered significant.

## 3 RESULTS

The mean age of children with normal and abnormal TCD was 8.4 ± 3.6 and 7.0 ± 3.5 years old (range: 2–15 years), respectively. Percentage SpO_2_ was significantly lower in children with abnormal TCD compared to normal TCD (*p* = 0.007) while WBC, MCV, MCH, and LDH were significantly higher in the abnormal TCD group than the normal TCD group (*p* < 0.01); the reticulocyte count, however, was not significant (*p* = 0.068) (Table 1). A multivariable regression that included all the significant variables, showed that low SpO_2_ (*p* = 0.033)_,_ increased WBC (*p* = 0.022), and high LDH level (*p* = 0.046) were significant determinants of abnormal TCD.

**TABLE 1:** Clinical and laboratory parameters of children with SCA

Parameters	Abnormal TCD	n	Normal TCD	n	*p*‐value
Age (years)	7.1 ± 3.6	69	8.4 ± 3.6	72	**0.030**
Gender (M/F)	42/30	69	36 /33	72	0.462
SpO_2_ (%)	94.4 ± 4.4	69	96.2 ± 3.1	70	**0.007**
WBC (×10^9^/L)	15.1 ± 5.1	69	12.8 ± 4.6	72	**0.005**
Hemoglobin (g/dl)	7.8 ± 1.1	69	8.1 ± 1.1	72	0.125
RBC (×10^9^/L)	2.8 ± 0.4	69	3.1 ± 0.7	72	**1.9 × 10^−4^**
MCV (fL)	84.5 ± 8.7	69	78.8 ± 8.9	72	**1.5 × 10^−4^**
MCH (pg)	28.0 ± 3.0	69	26.0 ± 3.3	72	**1.2 × 10^−4^**
Platelets (×10^9^/L)	464.2 ± 143.7	68	441.7 ± 143.9	69	0.337
Reticulocyte count (%)	6.6 ± 3.0	39	5.4 ± 2.5	35	0.068
HbF (%)	8.6 ± 4.8	51	7.6 ± 5.1	55	0.294
LDH (IU/L)	825.6 ± 395.1	51	638.7 ± 231.2	49	**0.005**

Note

Bold font depicts statistical significance at *p* < 0.05.

Abbreviations: HbF, fetal hemoglobin; LDH, Lactate dehydrogenase; MCH, Mean corpuscular hemoglobin; MCV, Mean corpuscular volume; RBC, Red blood cell; SpO_2_, hemoglobin oxygen saturation; TCD, Transcranial Doppler ultrasonography; WBC, white blood cell count.

The distribution of children having α‐thalassemia with heterozygous or homozygous deletion and those without α‐thalassemia trait in the abnormal and normal TCD groups was significantly different (*p* = 0.015). Frequency of α‐thalassemia trait in the children stratified as normal TCD was higher than those with abnormal TCD: 38 (52.8%) versus 21 (30.4%). The presence of α‐thalassemia decreased the risk of abnormal TCD velocities [Odds Ratio (OR) 0.39, 95% confidence interval (CI) 0.20–0.78, *p* = 0.007]. Children with α‐thalassemia trait showed a decreased risk of stroke in their first decade of life (OR: 0.34, 95% CI: 0.15–0.77, *p* = 0.009) (Table 2).

**TABLE 2:** Association of Alpha‐thalassemia and G6PD status with TCD

	Abnormal TCD n (%)	Normal TCD n (%)	OR (95% CI)	*p*‐value
α‐thalassemia status				
Without α‐thal trait	48 (69.6)	34 (47.2)		**0.015**
α‐thal with heterozygous deletion	19 (27.5)	30 (41.7)		
α‐thal with homozygous deletion	2 (2.9)	8 (11.1)		
Total α‐thal trait	21 (30.4)	38 (52.8)	0.39 (0.20 – 0.78)*	**0.007** *
Age classification				
≤ 10 years				
No α‐thal trait	39 (72.2)	24 (47.1)	0.34 (0.15 – 0.77)	**0.009**
α‐thal trait	15 (27.8)	27 (52.9)		
> 10 years				
No α‐thal trait	9 (60)	10 (47.6)	0.61 (0.16 – 2.32)	0.465
α‐thal trait	6 (40)	11 (52.4)		
G6PD status				
Normal	61 (88.4)	61 (84.7)	0.73 (0.27 – 1.93)	0.522
G6PDA‐	8 (11.6)	11 (15.3)		
Gender				
Male				
Normal	28 (77.8)	32 (76.2)	0.91 (0.32 – 2.64)	0.868
G6PDA‐	8 (22.2)	10 (23.8)		
Female				
Normal	33 (100)	29 (96.7)	‐	0.476^#^
G6PDA‐	‐	1 (3.3)		

Note: Bold fonts depict *p* < 0.05.

Abbreviations: G6PD, = Glucose‐6‐phosphate dehydrogenase; TCD:, Transcranial doppler ultrasonography; α‐thal ,= alpha‐thalassemia; Without α‐thal trait ,= α α/ α α; α‐thal with heterozygous deletion ,= α α/‐ α; α‐thal with homozygous deletion ,= ‐α/‐ α; α‐thal trait (children with at least a single deletion); OR,: odds ratio; CI,: confidence interval.

* OR (95%CI) and *p*‐value of total children with α‐thal trait and those without α‐thal trait.

# p‐value by Fisher’s exact test.

In a multivariable logistic regression that included age, SpO_2_, WBC, RBC, LDH, and α‐thalassemia, only SpO_2_ (OR: 0.87, 95% CI: 0.76–0.99, *p* = 0.033) and absence of α‐thalassemia (OR:4.48, 95% CI: 1.50–13.38, *p* = 0.007) were independently associated with abnormal TCD in children with SCA.

The frequency of G6PDA^−^ in children with abnormal and normal TCD was not significantly different (11.6% vs. 15.3%, *p* = 0.522). Classification of G6PD genotypes according to gender had no effect on the relationship between G6PDA^−^ and TCD and there was only one female (in the normal TCD group) with the presence of G6PDA^−^ (Table 2).

## 4 DISCUSSION

In this study, we found significantly lower hemoglobin oxygen saturation and high level of lactate dehydrogenase in children with abnormal TCD compared to those with normal TCD. Apart from lower age, a non‐modifiable biological factor, other modifiable significant hematological abnormalities observed in the abnormal TCD group include elevated WBC count, suggesting enhanced leukocytosis and elevated MCV, which may be indicative of higher osmotic fragility risk. These findings confirm our previous work in which elevated LDH and WBC count were linked to chronic hypoxia.^27^ Among Jamaican children with sickle cell anemia, Rankine‐Mullings et al^30^ also established significant associations of lower oxygen saturation, higher MCV, and leukocytosis with abnormal TCD. Involvement of sickle reticulocyte in endothelial activation and endothelial damage can be associated with the development of cerebral vasculopathy in children with SCA,^31^ and we observed high reticulocyte counts in a higher proportion of patients with abnormal TCD in the current study (6.6% vs. 5.4%; *p* = 0.068 – abnormal TCD vs. normal TCD, respectively). Our observation that abnormal TCD occurred at a higher frequency with younger age supports current recommendations for early TCD screening with the aim of initiating hydroxyurea therapy and chronic blood transfusion in children to limit the risk of strokes.^8^

In addition, we found a significantly lower frequency of alpha‐thalassemia trait among children having abnormal TCD compared with normal TCD. This finding is suggestive of a protective effect of the alpha‐thalassemia trait against abnormal TCD in Nigerian children with sickle cell anemia. These findings are supported by two independent studies from France in which the protective role of alpha‐thalassemia trait against abnormal TCD in children with sickle cell anemia was reported.^14^, ^26^ In both studies, the protective role of alpha‐thalassemia trait was linked to its association with reduced hemolysis among their sickle cell cohorts. Another mechanism that has been described is the association of alpha‐thalassemia trait with reduced exposure of phosphatidylserine on the surface of erythrocyte membrane, thereby reducing red cell adhesion to the endothelium and reduced vascular injury.^18^, ^32^ In contrast to our study, abnormal TCD measured according to STOP protocol did not show association with alpha‐thalassemia trait in the Tanzanian children with sickle cell anemia but confirmed protective effect of alpha‐thalassemia against abnormally low cerebral blood flow velocity.^15^ It is likely that the higher frequency of alpha‐thalassemia trait in the Tanzanian cohort, which is linked with the Bantu β‐globin haplotype accounts for the lower prevalence of abnormal TCD.^15^, ^33^ Meanwhile, higher prevalence of 10–16% abnormal TCD has been reported in other studies.^3^, ^14^

G6PD deficiency has been linked to increased accumulation of reactive oxygen species, depletion of glutathione stores, and enhanced oxidant stress, which in turn leads to decreased bioavailability of nitric oxide and vascular dysfunction.^19^ Previous studies had found no evidence that G6PD deficiency influences the severity of hemolysis or increases the incidence of acute anemic events in persons with SCA.^14^, ^34^ However, others have shown that the risk of an abnormal TCD was associated with increased G6PD deficiency.^14^, ^26^ We did not find an association between G6PDA^−^ and abnormal TCD in our cohort. Our finding is in agreement with the report of Belisario et al^25^ who also found that G6PD deficiency did not influence stroke risk or occurrence of ischemic stroke among Brazillian children with SCA. One major difference between our studies and the French cohorts is that G6PD deficiency was determined by G6PD enzyme activity and not by G6PDA‐ molecular deficiency presented in this study.^14^, ^26^ Also, most studies did not find an association of abnormal TCD/stroke with G6PD deficiency using G6PD enzyme activity or G6PDA^−^ variant.^24^, ^25^, ^35^, ^36^

Our study has some limitations. First, our sample size is small, and it is possible that a larger study would provide better power to detect associations. Secondly, the study was a single‐center design which may limit the generalization of the findings, but the Sickle Cell Foundation Lagos offers clinical services to patients referred from primary, secondary, and tertiary clinics in Lagos, Nigeria. Thirdly, only single and double 3.7 kb α‐globin gene deletions were investigated in this study. Besides, we assessed only G6PD deficiency (A^−^) and did not examine other forms of G6PD polymorphisms that may be present in our population. Large cohorts will be needed in future studies to validate the relationship between TCD and alpha‐thalassemia trait/G6PD deficiency.

## CONFLICT OF INTEREST

The authors report no conflict of interest.

## Data Availability

The data that support the findings of this study are available from the corresponding author upon reasonable request.
